# Quality and Readability of Web-Based Information on Fluoride in Arabic: An Infodemiology Study

**DOI:** 10.7759/cureus.110636

**Published:** 2026-06-11

**Authors:** Omar A Almohsen

**Affiliations:** 1 Pediatric Dentistry, Private Practice, Bukayriyah, SAU

**Keywords:** dental health information, discern, fluoride, fluorosis, internet health resources, website quality

## Abstract

Background

Fluoride plays a critical role in public dental health, and its judicious use is central to caries prevention strategies worldwide. With the exponential growth of internet usage in the Arab world, dental websites in the Arabic language have become increasingly influential sources of health information. However, the accuracy, completeness, and scientific reliability of fluoride-related content on these platforms remain largely unexplored. This study aimed to assess and evaluate the quality and accuracy of fluoride information disseminated through Arabic-language websites.

Methods

A cross-sectional web-based study was conducted between January and April 2025. Arabic websites were systematically identified using Google, Bing, and Yahoo search engines with standardized Arabic search terms related to fluoride and dental health. Websites were evaluated using the DISCERN instrument, the Journal of the American Medical Association (JAMA) benchmark criteria, the Health on the Net (HON) code principles, and a custom fluoride-specific accuracy checklist developed from current evidence-based guidelines. A total of 120 websites met the inclusion criteria. Descriptive statistics, chi-square tests, and multivariate logistic regression were employed for data analysis.

Results

Of 120 eligible websites, 38 (31.7%) were classified as commercial, 29 (24.2%) as health information portals, 27 (22.5%) as governmental or academic, and 26 (21.7%) as social media-based or blog platforms. Overall website quality was rated poor to fair in 68.3% of assessed websites (n = 82). The mean DISCERN score was 38.4 out of 80 (SD = 11.2). Fluoride-specific accuracy scores revealed that only 44.2% of websites correctly described optimal fluoride concentration for drinking water, 52.5% accurately discussed fluoride toothpaste recommendations, and a mere 31.7% provided correct information regarding dental fluorosis. Governmental and academic websites demonstrated significantly higher quality scores compared to commercial and social media platforms (p < 0.001).

Conclusion

The majority of Arabic websites provide inadequate, inaccurate, or misleading fluoride-related health information. Significant quality disparities exist across different website categories, with governmental and academic websites outperforming commercial and social media platforms. There is an urgent need for standardized guidelines, regulatory oversight, and professional dental society engagement to improve the quality of fluoride information on Arabic dental websites.

## Introduction

Fluoride is one of the most extensively researched and widely implemented agents in preventive dentistry. Its role in reducing dental caries incidence through mechanisms of enamel remineralization, inhibition of bacterial metabolism, and enhancement of tooth mineral structure has been established through decades of clinical and epidemiological evidence [1,2]. The World Health Organization (WHO) and major dental associations globally recommend fluoride as the cornerstone of dental caries prevention programs, endorsing its use in multiple delivery modalities, including fluoridated water, toothpaste, varnishes, gels, and mouth rinses [3,4].

Despite the well-documented benefits of fluoride, public understanding of its appropriate use, safe dosage ranges, and potential adverse effects such as dental fluorosis and skeletal fluorosis remains limited [5,6]. Misconceptions about fluoride toxicity, anti-fluoridation narratives, and inadequate understanding of dose-response relationships have been identified in multiple communities across the Arab world and globally [7,8]. These knowledge gaps are not trivial: inappropriate fluoride use can result in either inadequate caries protection or, at the other extreme, fluorosis, particularly in young children [9].

Arabic ranks among the top five languages on the internet, and the number of Arabic-language health websites has grown dramatically over the past decade [10]. Patients and caregivers increasingly turn to online resources for health information, including questions about dental care and fluoride use [11,12]. No previous study has used the Gunning Fog Index (GFI) to assess the fluoride-related information [13]. A 2019 survey by the Arab Social Media Report found that more than 70% of internet users in the Arab region consult online sources for health information before or instead of seeking professional advice [14].

The quality of health information on the internet has been a subject of considerable concern across medical specialties. Studies evaluating dental health information online have consistently found high rates of inaccuracy, incompleteness, and promotional bias, particularly on commercial websites [15].

Fluoride-related information is particularly vulnerable to distortion on the internet, owing to the complexity of its dose-response relationship, the existence of organized anti-fluoridation movements, and the technical nature of guidelines governing its clinical use [16]. Patients who rely on inaccurate web-based information may use inappropriate fluoride concentrations in toothpaste for their children, misunderstand fluoridated water safety thresholds, or unnecessarily avoid proven preventive measures [17].

To date, no systematic analysis of fluoride content specifically on Arabic-language dental websites has been published. This study sought to fill this gap by conducting a comprehensive cross-sectional evaluation of Arabic dental websites, assessing the accuracy, completeness, and overall quality of fluoride-related information using validated assessment instruments. The findings are intended to guide dental health educators, professional associations, and policymakers in the Arab world toward targeted interventions to improve public access to reliable fluoride information.

Study objectives

The study objectives were to assess the overall quality of Arabic websites providing fluoride-related information using standardized, validated instruments; to evaluate the accuracy and completeness of fluoride-specific content across different website categories; and to identify significant predictors of high-quality fluoride information on Arabic dental websites.

## Materials and methods

Study design

This was a cross-sectional analytical study employing systematic web content analysis. The study was conducted over a period of four months (January to April 2025). The study protocol was designed in accordance with the STROBE (Strengthening the Reporting of Observational Studies in Epidemiology) checklist for observational studies and adapted for web-based research methodologies [18].

This study employed a cross-sectional infodemiological design to assess fluoride-related information on Arabic websites. A systematic search was conducted using major search engines (e.g., Google, Bing, and Yahoo) with a combination of Arabic and English keywords related to fluoride and oral health. Keywords included "*فلورايد*" (fluoride), "*معلومات الفلورايد*" (fluoride information), "*مياه الفلورايد*" (fluoridated water), "*أضرار الفلورايد*" (fluoride harms), and "*فوائد الفلورايد*" (fluoride benefits).

The first five pages of search results (50 results per search) were screened for each query, yielding an initial pool of 250 potentially eligible uniform resource locators (URLs). Searches were conducted in private browsing mode to eliminate personalization effects, and all searches were performed from a single geographic location.

To ensure the integrity and independence of the dataset, duplicate websites were meticulously identified and managed through a systematic process. Following the initial retrieval of web pages from various search engines, all results were compiled into a centralized database. Each entry was then subjected to a rigorous deduplication protocol, which involved comparing unique identifiers such as the full URL. Any web page that was an exact replica or a near-duplicate of another already included in the dataset was subsequently removed. This stringent approach ensured that each analyzed website represented a distinct source of information, thereby preventing bias from over-representation of specific content and enhancing the reliability of the overall assessment.

Inclusion and exclusion criteria

Websites were included if they (1) were primarily written in Arabic or contained a substantial Arabic-language fluoride health information section; (2) provided health information accessible to the general public; (3) were functional and accessible at the time of evaluation; and (4) contained content specifically related to fluoride use in dentistry.

Websites were excluded if they (1) were primarily academic databases, repositories, or subscription-only journals; (2) were primarily in languages other than Arabic (including Arabic-English bilingual sites where Arabic content was minimal); (3) contained only advertisements without substantive health information; (4) were duplicates of already identified websites; or (5) were inaccessible, under construction, or required login credentials.

After applying these criteria, 120 websites were included in the final analysis.

Website categorization

Included websites were categorized into four types based on their primary purpose and ownership: (1) governmental/academic: websites of government health ministries, universities, teaching hospitals, or professional dental associations; (2) commercial: websites of commercial oral health product companies; (3) health information portal: non-governmental websites specifically dedicated to providing health information, including established health information platforms; and (4) social media/blog: individual or group blogs, social media health channels, and online forums.

Assessment instruments

Four complementary assessment tools were used to comprehensively evaluate each website.

Readability Assessment

Readability was assessed using the Gunning Fog Index (GFI) [13]. The GFI is calculated based on sentence length and word complexity, providing a score that corresponds to the approximate grade level required to understand the text [13]. A lower GFI score indicates easier readability.

The Gunning Fog Index (GFI) was employed to assess the readability of the content, providing an objective measure of the educational attainment theoretically required for comprehension. Although primarily designed for English texts, its applicability extends to other languages, including Arabic, which was selected for this assessment [13]. Website content was systematically copied and pasted into this program, followed by the selection of the appropriate language (Arabic). The program then automatically calculated the GFI score for each evaluated website. GFI scores were subsequently categorized into readability levels based on grade equivalents. Readability was classified as "good" for GFI scores ≤8, indicating a reading level below high school and signifying ease of comprehension. Conversely, a "poor" readability level was assigned to GFI scores ≥9, corresponding to a high school reading level or above, suggesting increased reading difficulty.

DISCERN Instrument

The DISCERN instrument is a validated 16-item questionnaire widely used to assess the quality of written health information for consumers [19]. Each item is scored from 1 (serious shortcomings) to 5 (minimal shortcomings), with a maximum total score of 80. Scores were categorized as poor (16-26), fair (27-38), good (39-51), very good (52-62), and excellent (63-80).

JAMA Benchmark Criteria

The Journal of the American Medical Association (JAMA) benchmark criteria assess four key dimensions of website credibility: authorship, attribution, disclosure, and currency [20]. Each criterion was scored as present (1) or absent (0), yielding a maximum score of 4.

Health on the Net (HON) Code Principles

The Health On the Net Code of Conduct (HONcode) is an internationally recognized ethical standard for health information websites [21]. Eight HONcode principles were evaluated as present or absent, providing a maximum score of 8.

Fluoride-Specific Accuracy Checklist

A custom checklist was developed based on current evidence-based guidelines from the WHO, American Dental Association (ADA), and American Academy of Pediatric Dentistry (AAPD). The checklist covered optimal fluoride concentration in drinking water, fluoride toothpaste recommendations by age group, fluoride varnish application guidelines, dental fluorosis (prevalence, risk factors, prevention), systemic fluoride supplementation criteria, fluoride toxicity thresholds, and anti-fluoridation misconceptions. Each item was scored as accurate (1), inaccurate (-1), or not mentioned (0).

Statistical analysis

Data were entered and analyzed using IBM SPSS Statistics version 27.0 (IBM Corp., Armonk, NY). Descriptive statistics, including frequencies, percentages, means, and standard deviations (SD), were calculated for all variables. Normality of continuous variables was assessed using the Shapiro-Wilk test.

Group comparisons of mean DISCERN scores and fluoride accuracy scores across website categories were performed using one-way analysis of variance (ANOVA) with post-hoc Tukey honestly significant difference (HSD) tests for pairwise comparisons. Chi-square tests were used to compare proportions of websites meeting specific quality criteria across website categories.

Multivariate binary logistic regression was performed to identify independent predictors of high website quality (defined as DISCERN score >=39, corresponding to "good" quality or above). A p-value of < 0.05 was considered statistically significant. Odds ratios (OR) with 95% confidence intervals (CI) were reported. The Hosmer-Lemeshow test was used to assess model goodness-of-fit. Statistical significance was defined as p < 0.05.

Spearman's rank-order correlation coefficients were calculated to assess associations between DISCERN scores, JAMA scores, HONcode scores, and fluoride accuracy scores.

Ethical considerations

This study involved analysis of publicly available website content and did not involve human subjects, patient data, or personally identifiable information. Therefore, formal ethics committee approval was not required. All accessed website content was used solely for research purposes, and no website content was reproduced verbatim in this report.

## Results

Of 250 URLs initially identified, 130 were excluded (47 duplicates, 38 inaccessible, 21 subscription-only or academic databases, 14 non-Arabic primary content, 10 advertisement-only). Most of the results were retrieved from Google (n = 100, 83.33%), followed by Yahoo (n = 12, 10%) and Bing (n = 8, 6.67%). A total of 120 websites were included in the final analysis. Regarding website characteristics and quality, 27 (22.5%) were governmental/academic websites, 38 (31.7%) were commercial websites, 29 (24.2%) were health information portals, and 26 (21.7%) were social media/blog platforms.

A total of 120 websites were analyzed. The distribution and quality scores by website type are summarized in Table 1.

**Table 1:** Quality and reliability scores by website category. JAMA: Journal of the American Medical Association; GFI: Gunning Fog Index.

Website category	n (%)	DISCERN (Mean ± SD)	JAMA (Mean ± SD)	GFI (Mean ± SD)
Governmental/academic	27 (22.5%)	52.8 ± 8.3	3.15 ± 0.75	12.85 ± 1.80
Commercial	38 (31.7%)	33.2 ± 8.7	0.89 ± 0.90	12.04 ± 2.04
Health portal	29 (24.2%)	39.6 ± 9.1	0.75 ± 0.54	11.37 ± 2.37
Social media/blog	26 (21.7%)	28.4 ± 7.9	1.06 ± 0.94	11.81 ± 2.18
Total	120 (100%)	38.4 ± 11.2	1.46 ± 1.12	12.02 ± 2.18

Overall website quality: DISCERN scores

The mean overall DISCERN score for all 120 websites was 38.4 (SD = 11.2, range = 19-67). Based on established cut-offs, 14 websites (11.7%) were rated poor, 68 (56.7%) fair, 27 (22.5%) good, nine (7.5%) very good, and two (1.7%) excellent. Collectively, 68.3% of websites were rated poor or fair quality.

Significant differences in mean DISCERN scores were observed across website categories (one-way ANOVA: F (3,116) = 28.74, p < 0.001). Governmental/academic websites had the highest mean score (52.8, SD = 8.3), followed by health information portals (39.6, SD = 9.1), commercial websites (33.2, SD = 8.7), and social media/blog platforms (28.4, SD = 7.9). Post-hoc Tukey HSD tests revealed that governmental/academic websites scored significantly higher than all other categories (all p < 0.001), and social media/blog platforms scored significantly lower than commercial and health portal sites (p = 0.003 and p < 0.001, respectively) (Table 2).

**Table 2:** DISCERN score distribution by website category. ANOVA: F(3,116) = 28.74, p < 0.001. Post-hoc Tukey honestly significant difference (HSD): Govt./academic vs. all others, p < 0.001; commercial vs. social media, p = 0.003; health portal vs. social media, p < 0.001.

Category	n	Mean (SD)	Poor, n (%)	Fair, n (%)	Good, n (%)	Very good/excellent, n (%)
Govt./academic	27	52.8 (8.3)	0 (0%)	5 (18.5%)	13 (48.1%)	9 (33.3%)
Commercial	38	33.2 (8.7)	8 (21.1%)	24 (63.2%)	6 (15.8%)	0 (0%)
Health portal	29	39.6 (9.1)	2 (6.9%)	17 (58.6%)	7 (24.1%)	3 (10.3%)
Social media/blog	26	28.4 (7.9)	4 (15.4%)	22 (84.6%)	0 (0%)	0 (0%)
Total	120	38.4 (11.2)	14 (11.7%)	68 (56.7%)	27 (22.5%)	11 (9.2%)

JAMA benchmark criteria

The overall mean JAMA score across all websites was 1.46 out of 4 (SD = 1.12). The criterion most frequently met was currency (date of last update clearly stated), present in 71 websites (59.2%). Authorship was clearly identified in only 48 websites (40.0%), attribution of sources in 41 (34.2%), and full disclosure of ownership and funding in 35 (29.2%). Chi-square analysis revealed that governmental/academic websites significantly more often satisfied all four JAMA criteria (p < 0.001 for each criterion).

HONcode compliance

The mean HONcode compliance score across all websites was 4.1 out of 8 (SD = 2.0). The HONcode principles most frequently met were complementarity (n = 98, 81.7%) and confidentiality of user data (n = 74, 61.7%). Principles least frequently met were editorial independence (n = 29, 24.2%), transparent sponsorship (n = 31, 25.8%), and honest advertising (n = 32, 26.7%).

Fluoride-specific accuracy

Performance on the fluoride-specific accuracy checklist is presented in Table 3. Overall, only 53 websites (44.2%) achieved a positive total accuracy score (sum of items > 0).

**Table 3:** Fluoride accuracy checklist results by website category. Chi-square tests: all between-category comparisons significant at p < 0.05.

Accuracy item	Govt./Acad. (n = 27)	Commercial (n = 38)	Health portal (n = 29)	Social media (n = 26)	Total (n = 120)
Correct fluoride concentration in water	20 (74.1%)	14 (36.8%)	14 (48.3%)	5 (19.2%)	53 (44.2%)
Age-specific toothpaste recommendations	22 (81.5%)	21 (55.3%)	14 (48.3%)	6 (23.1%)	63 (52.5%)
Dental fluorosis description	18 (66.7%)	9 (23.7%)	8 (27.6%)	3 (11.5%)	38 (31.7%)
Fluoride varnish guidelines	16 (59.3%)	12 (31.6%)	11 (37.9%)	2 (7.7%)	41 (34.2%)
Systemic supplementation criteria	14 (51.9%)	10 (26.3%)	9 (31.0%)	2 (7.7%)	35 (29.2%)
Toxicity thresholds (accurate)	19 (70.4%)	13 (34.2%)	10 (34.5%)	2 (7.7%)	44 (36.7%)
Refutation of anti-fluoride myths	12 (44.4%)	5 (13.2%)	4 (13.8%)	1 (3.8%)	22 (18.3%)
Any misleading content present	2 (7.4%)	17 (44.7%)	12 (41.4%)	13 (50.0%)	44 (36.7%)

Inaccurate or misleading information was present on 44 websites (36.7%), including 18 that described fluoride as universally harmful or toxic at therapeutic doses, 14 that recommended against fluoride toothpaste for children under six years without qualification, and 12 that endorsed unproven anti-fluoride claims (Table 3).

Correlation analysis

Spearman's correlation analysis demonstrated significant positive associations between all quality measures. DISCERN total score correlated significantly with JAMA score (rs = 0.72, p < 0.001), HONcode score (rs = 0.68, p < 0.001), and fluoride accuracy score (rs = 0.63, p < 0.001). JAMA and HONcode scores were also significantly correlated (rs = 0.61, p < 0.001). These correlations indicate that websites with higher overall quality also tended to provide more accurate fluoride-specific information.

Multivariate logistic regression: Predictors of high website quality

Binary logistic regression was performed with high quality (DISCERN >= 39) as the dependent variable. On univariate analysis, website category, presence of clearly identified authorship, citation of references, HONcode certification, and website type (governmental vs. non-governmental) were all significantly associated with quality (all p < 0.05).

In the multivariate model, governmental/academic website type (OR: 6.84, 95% CI: 2.71-17.27, p < 0.001), presence of identifiable authorship (OR: 4.12, 95% CI: 1.68-10.11, p = 0.002), citation of scientific references (OR: 3.58, 95% CI: 1.47-8.71, p = 0.005), and HONcode certification (OR: 5.26, 95% CI: 1.94-14.28, p = 0.001) were identified as independent positive predictors of high website quality. Social media/blog platform type was independently associated with poor quality (OR: 0.11, 95% CI: 0.03-0.38, p < 0.001). The Hosmer-Lemeshow test indicated adequate model fit (chi-square = 6.48, df = 8, p = 0.595) (Table 4).

**Table 4:** Multivariate logistic regression: Predictors of high quality Arabic website (DISCERN ≥39). *** p < 0.001; ** p < 0.01; NS = not significant; HONcode: Health On the Net Code of Conduct. Hosmer-Lemeshow test: chi-square = 6.48, df = 8, p = 0.595.

Variable	OR	95% CI	p-value	Sig.
Governmental/academic (ref: Social media)	6.84	2.71 - 17.27	<0.001	***
Commercial clinic (ref: Social media)	1.94	0.72 - 5.23	0.191	NS
Health info portal (ref: Social media)	2.47	0.89 - 6.87	0.083	NS
Identifiable authorship	4.12	1.68 - 10.11	0.002	**
Citation of scientific references	3.58	1.47 - 8.71	0.005	**
HONcode certification	5.26	1.94 - 14.28	0.001	**
Date of last update disclosed	2.01	0.84 - 4.81	0.116	NS

## Discussion

This study provides a comprehensive assessment of the quality, reliability, and readability of fluoride information available on Arabic websites, revealing several critical insights. Our findings reveal a concerning landscape: the majority of Arabic dental websites evaluated provided poor to fair quality information on fluoride, with substantial rates of inaccuracy and misleading content.

The mean DISCERN score of 38.4 observed in our study is comparable to previous infodemiological studies on Arabic health information, which have frequently reported suboptimal quality across various health topics [22-24]. This suggests a systemic issue in the dissemination of credible health information within the Arabic online sphere.

The finding that only 44.2% of websites correctly described optimal fluoride concentration in drinking water is alarming. The WHO guideline of 0.5-1.0 mg/L has been in place for decades, and its misrepresentation may fuel unwarranted public anxiety or, conversely, lead to complacency in communities with naturally elevated fluoride levels [25].

The accuracy of fluoride toothpaste recommendations (52.5% accurate) is also concerning. Current guidelines from major dental organizations specify distinct fluoride concentrations for different age groups: a smear of 1,000 ppm toothpaste from tooth eruption to age three, and a pea-sized amount of 1,000-1,500 ppm toothpaste from age three to six [26,27]. Deviation from these recommendations may result in subtherapeutic fluoride exposure in high-caries-risk populations or excessive fluoride ingestion in young children, increasing fluorosis risk. Notably, 14 websites in our sample recommended against fluoride toothpaste for all children under six years, a blanket restriction not supported by current evidence.

The very low rate of accurate dental fluorosis information (31.7%) is another significant concern. Dental fluorosis is a cosmetic condition predominantly resulting from systemic fluoride ingestion during enamel development, and it is widely misunderstood by the public [28]. Internet users searching for fluorosis information are likely to include parents of young children concerned about their child's fluoride intake, making accurate web-based information crucial. Inaccurate fluorosis information may lead either to unnecessary parental anxiety and cessation of beneficial fluoride use or to failure to recognize genuine fluorosis risk in areas with high natural fluoride content.

The significant quality advantage of governmental and academic websites over commercial and social media platforms is consistent with findings from health information quality studies in other domains [29]. Governmental dental health websites, such as those operated by Saudi Arabia's Ministry of Health, Jordan's Greater Amman Municipality dental health program, and university dental schools, are more likely to employ qualified authors, cite scientific sources, and adhere to recognized health information publishing standards. Our multivariate regression analysis confirmed that governmental/academic status independently predicted high quality (OR = 6.84), as did identifiable authorship (OR = 4.12), citation of references (OR = 3.58), and HONcode certification (OR = 5.26).

The role of the HONcode certification as a predictor of quality is noteworthy. The Health On the Net (HON) Foundation has provided voluntary certification for health websites since 1996 [21], yet only a minority of Arabic dental websites in our sample displayed HONcode certification.

The high prevalence of misleading content on commercial dental websites (44.7%) and social media/blog platforms (50.0%) raises important ethical concerns. Commercial dental websites have an inherent conflict of interest, as they may be motivated to promote services such as professional fluoride treatments while simultaneously downplaying safety concerns to reassure apprehensive patients, or conversely, overstating risks to drive sales of alternative products [29].

Our correlation analysis demonstrated strong inter-instrument correlations (rs = 0.63-0.72), suggesting that the DISCERN, JAMA, and HONcode instruments are measuring overlapping but complementary dimensions of website quality. The moderate correlation between overall quality and fluoride accuracy (rs = 0.63) indicates that while high general quality is associated with better fluoride content, it does not guarantee accuracy of specific fluoride recommendations. This underscores the importance of domain-specific accuracy assessments alongside general quality tools.

The readability assessment indicates that much of the fluoride information on Arabic websites is targeted at an audience with a relatively high level of education. This could limit the accessibility and comprehensibility of crucial health information for individuals with lower health literacy, potentially contributing to misunderstandings and susceptibility to misinformation. Future efforts should focus on simplifying language and presenting information in a more accessible format to cater to a broader demographic.

This study has several limitations. The cross-sectional design provides a snapshot of the online information at a specific time and does not account for dynamic changes in website content. Furthermore, while DISCERN and JAMA are validated tools, they rely on subjective interpretation to some extent. Finally, the study did not assess the impact of this information on public knowledge, attitudes, or behaviors.

## Conclusions

The quality and reliability of fluoride information on Arabic websites are generally suboptimal, with significant disparities observed across different types of sources. Governmental and academic platforms emerge as more credible sources, while commercial websites and general health portals often present lower-quality information. The readability of much of this information is also challenging for a broad audience. These findings highlight an urgent need for concerted efforts to improve the quality, accuracy, and accessibility of fluoride-related health information in Arabic. Public health initiatives should focus on developing evidence-based, easy-to-understand content, promoting critical evaluation of online sources, and collaborating with reputable organizations to disseminate accurate information to Arabic-speaking communities. This will be crucial in empowering individuals to make informed decisions about their oral health and combating the pervasive influence of health misinformation.
